# Comparative Transcriptome Analysis of Gene Expression and Regulatory Characteristics Associated with Different Vernalization Periods in *Brassica rapa*

**DOI:** 10.3390/genes11040392

**Published:** 2020-04-05

**Authors:** Yun Dai, Shujiang Zhang, Xiao Sun, Guoliang Li, Lingyun Yuan, Fei Li, Hui Zhang, Shifan Zhang, Guohu Chen, Chenggang Wang, Rifei Sun

**Affiliations:** 1 Institute of Vegetables and Flowers, Chinese Academy of Agricultural Sciences, Beijing 100081, China; dy1027@ahau.edu.cn (Y.D.); zhangshujiang@caas.cn (S.Z.); 13121273253@163.com (X.S.); liguoliang@caas.cn (G.L.); lifei@caas.cn (F.L.); zhanghui05@caas.cn (H.Z.); zhangshifan@caas.cn (S.Z.); 2 Vegetable Genetics and Breeding Laboratory, College of Horticulture, Anhui Agricultural University, Hefei 230036, China; yuanlingyun@ahau.edu.cn (L.Y.); cgh@ahau.edu.cn (G.C.)

**Keywords:** *Brassica rapa*, enrichment pathway, self-organizing feature map, transcriptome analysis, vernalization

## Abstract

*Brassica rapa* is an important Chinese vegetable crop that is beneficial to human health. The primary factor affecting *B. rapa* yield is low temperature, which promotes bolting and flowering, thereby lowering its commercial value. However, quickened bolting and flowering can be used for rapid breeding. Therefore, studying the underlying molecular mechanism of vernalization in *B. rapa* is crucial for solving production-related problems. Here, the transcriptome of two *B. rapa* accessions were comprehensively analyzed during different vernalization periods. During vernalization, a total of 974,584,022 clean reads and 291.28 Gb of clean data were obtained. Compared to the reference genome of *B. rapa*, 44,799 known genes and 2280 new genes were identified. A self-organizing feature map analysis of 21,035 differentially expressed genes was screened in two *B. rapa* accessions, ‘Jin Wawa’ and ‘Xiao Baojian’. The analysis indicated that transcripts related to the plant hormone signal transduction, starch and sucrose metabolism, photoperiod and circadian clock, and vernalization pathways changed notably at different vernalization periods. Moreover, different expression patterns of *TPS*, *UGP*, *CDF*, *VIN1*, and seven hormone pathway genes were observed during vernalization between the two accessions. The transcriptome results of this study provide a new perspective on the changes that occur during *B. rapa* vernalization, as well as serve as an excellent reference for *B. rapa* breeding.

## 1. Introduction

The vegetative and reproductive growth stage are important stages in the life cycle of land plants. After a period of vegetative growth, land plants undergo reproductive growth, which is regulated by various environmental factors and endogenous signals that induce flowering. This transformation process is called floral induction, also known as flowering transition. A regulatory network ensures that plants accumulate sufficient vegetative growth over time, transforming them into reproductive growth [1]. With the development of molecular biology, five pathways that influence bolting and flowering in rice, wheat, and *Arabidopsis* have been identified, including the photoperiod, gibberellin (GA), vernalization, autonomous, and age pathways [2,3,4,5,6].

Typically, vernalization and long periods of daylight are the main factors that drive plant vegetative growth to reproductive development [7,8]. Thus far, vernalization and long periods of daylight have been found to regulate flowering factors, including the flowering promoter (*FT*) and related interaction genes that affect the flowering time [9]. In *Arabidopsis thaliana*, the vernalization pathway molecular mechanism regulates flower locus C (*FLC*), frigida (*FRI*), vernalization 1 (*VRN1*), vernalization 2 (*VRN2*), and vernalization insensitive 3 (*VIN3*). *FRI* promotes the high expression of *FLC* and the low expression of *FT*, which leads to flowering delays [10]. *VRN1*, *VRN2*, and *VIN3* are negative regulators of *FLC*, of which, *VRN2* was the first vernalization gene to be cloned [11]. *VRN1* and *VRN2* are expressed in different plant tissues and developmental stages. They are not induced by vernalization, but control the low expression of *FLC* after vernalization has concluded [12,13]. *VIN3* is induced by vernalization, participates in *FLC* chromatin remodeling, and plays a suppressive role [14]. In monocotyledonous wheat, the genes necessary for vernalization include *VRN1*, *VRN2*, and vernalization 3 (*VRN3*), none of which are homologues to the *VRNs* of *Arabidopsis* and do not contain *FLC-like* [15,16]. Both *VRN1* and *VRN3* in Arabidopsis have homology genes *FUL* and *FT* in wheat [15,17]. *VRN2* possesses no homologous genes in *Arabidopsis* and is a negative regulator of flowering in wheat during unvernalized [18]. Differences in the vernalization pathways between *Arabidopsis* and monocotyledons may be due to the independent evolution of their response mechanisms to vernalization.

*Brassica rapa* is a leafy vegetable that belongs to the *Brassicaceae* family. It originated in China and has been widely distributed throughout the country, accounting for a wide area of vegetable cultivation. *B. rapa* is a high-yielding vegetable, is durable to storage and transportation, possesses a long supply period, is rich in nutrition, and diverse in consumption methods. This plant also occupies the primary position in vegetable production and daily consumption in China. It is generally planted in the fall from August to November. Spring planting was later developed to meet the demand for supply throughout the year. Spring sowing occurs under low temperature conditions and periods of long daylight, which cause *B. rapa* to prematurely bolt and flower, thereby causing the plant to lose some of its commercial value [18]. However, quickened bolting is a rapid breeding method that can help produce better accessions faster. Therefore, low-temperature vernalization has some positive effects on the cultivation of *B. rapa*, but further research is required to verify these effects.

In this study, a 50-day vernalization experiment was conducted using two *B. rapa* accessions. Samples with different vernalization times were selected according to their flowering times for transcriptome analysis. The expression of differentially expressed genes (DEGs) in the plant hormone signal transduction, starch and sucrose metabolism, photoperiod and circadian clock, and vernalization pathways were analyzed during two vernalization processes. The purpose of this study was to clarify the effects of different vernalization periods on the two different bolting *B. rapa* accessions.

## 2. Materials and Methods

### 2.1. Plant Materials andTreatments

Two *B. rapa* accessions, 200 late-bolting ‘Jin Wawa’ (JWW) and 200 early-bolting ‘Xiao Baojian’ (XBJ), were grown in a nursery greenhouse at the Chinese Academy of Agricultural Sciences in Beijing, China (39°56′ N, 116°20′ E), which consisted of highly inbred lines. Plants were initially grown at 25 ± 2 °C under natural light for 32 days. Afterwards, 100 ‘JWW’ and 100 ‘XBJ’ were moved into a vernalization room at 4 °C under a 16/8 h light/dark photoperiod and 150 μmol m^−2^ s^−1^ light intensity. Plants were randomly arranged. The other 100 ‘JWW’ and 100 ‘XBJ’ were moved into a climate chamber at 25 °C under the same conditions as the vernalization room. The day when two *B. rapa* accessions transferred into the vernalization room and the climate chamber were recorded as 0days, and the vernalization and control were treated for a total of 50 days, with each 5 days being considered a period. For the time of day after treatment (DAT), the default DAT conditions were 4 °C for the vernalization treatment days and 25 °C DAT for the control treatment days. The third fully expanded leaves from the center of the plants were sampled every 5 days from both treatments for a total of 50 days. Three biological replicates of the leaves were randomly collected from each individual seedling. Each treatment sample was collected separately, immediately frozen in liquid nitrogen, and stored at −80 °C for future transcriptome sequencing (i.e., RNA-Seq) and quantitative real-time polymerase chain reaction (qRT-PCR) analyses. After random sampling during each period, eight seedlings from the previous 4 °C treatment and grown in a nursery greenhouse (25 ± 2 °C under natural light). The flowering times were recorded, which began when the first petal was fully expanded. 

### 2.2. RNA Extraction and Illumina Sequencing

Total RNA was extracted from leaf samples using an RNAprep Pure Plant kit (Tiangen, Beijing, China) following the manufacturer’s instructions. RNA concentrations and quality were verified using an Agilent 2100 Bioanalyzer (Agilent Technologies, Santa Clara, CA, USA). A total amount of 1 μg RNA per sample was used as input material for the RNA sample preparations. Forty-two cDNA libraries (2 accessions ×7 periods ×3 replicates) were constructed and sequenced using NEBNext® Ultra™ RNA Library Prep Kit for Illumina (New England Biolabs, Ipswich, MA, USA) following manufacturer’s recommendations. mRNA was purified from total RNA using poly-T oligo-attached magnetic beads. cDNA fragments of preferentially 240 bp in length were selected, the library fragments were purified with AMPure XP system (Beckman Coulter, Brea, CA, USA).Each library generated > 6 GB raw data. Raw reads were processed in fastq format. The adaptor sequences and low-quality sequence reads were removed from the data sets, including reads that removed more than 10% of N and more than 50% of reads with a base of Q ≤ 10. The raw data have been submitted under BioProject number PRJNA615255 to the Sequence Read Archive (SRA) database at NCBI.

### 2.3. Transcriptome Analysis

The *B. rapa* reference genome sequence and annotation files were downloaded from the Brassica Database v3.0 [19]. Clean reads were mapped to the *B. rapa* reference genome sequence using Tophat v2.0.9 [20]. Cufflinks v2.2.1 was used to assemble reference annotation files, estimate the levels of unigene expression, and normalize the relative abundance of each transcript in each sample to fragments per kilobase of exon per million fragments mapped (FPKM) [21]. DESeq R v1.10.1 was used for processing to detect transcriptional changes in ‘JWW’ and ‘XBJ’ during vernalization [22]. DEGs were identified by comparing pairs of samples in the two sampling points (J1-J2, J1-J3, J1-J4, J1-J5, J1-J6, JCK-J4, X1-X2, X1-X3, X1-X4, X1-X5, X1-X6, XCK-X4) by using the DESeq R package. In addition, the false discovery rate (FDR)< 0.01 & |log2 (fold change)| ≥ 2 was set as the threshold for significantly differential expression. *p*-values of the FDR were controlled using Benjaminie and Hodgberger’s method; genes with a *p*-value < 0.01 were considered differentially expressed.

### 2.4. Self-Organizing Feature MapAnalysis

A self-organizing feature map (SOM) is a data matrix and visualization method based on neural networks [23]. For the treatment methods of this experiment, SOM was used to design a formula containing the condition factor, time factor, and interaction between these factors. ‘JWW’ and ‘XBJ’ DEGs were processed, normalized, and used in SOM machine learning for centralizing and clustering. Through this analysis, 21,035 DEGs were identified between ‘JWW’ and ‘XBJ’. 21305 DEGs (Table S1) was normalized by using R language _scale, which is beneficial for the next step of SOM cluster analysis. The SOM approach was used to find a set of central points and map each object in the dataset to the corresponding central point under the most similar principle [24].

In neural network terminology, each neuron corresponds to a central point. The parameters for the SOM analysis were set as follows: som_model< -som (data_train_matrix, grid = somgrid (xdim = 30, ydim = 30, topo = “hexagonal”), rlen = 100, alpha = c (0.05, 0.01), keep.data = TRUE). The SOMgrid defines the size of the network as 30 × 30 with six sides arranged between the squares. A matrix of the gene-to-sample expression values was used as input data. Genes with similar expression values were self-organized into the hexagon vicinity. The resulting map was visualized to show time-specific gene expression, in which a center point simplicity set defined multiple clusters that were grouped into the same cluster as the object closest to the center point. The expression model clustering was divided into each of the 20 ‘JWW’ and ‘XBJ’ clusters.

### 2.5. Gene Annotations andqRT-PCR

Gene ontology (GO) enrichment analysis was conducted on the DEGs using GOseq R software [25]. KOBAS software was used to perform DEG enrichment statistics in the KEGG pathway [26]. Brassica Database v3.0 was used as reference lists [19]. Twelve DEGs were randomly selected to confirm the transcriptome data by qRT-PCR analysis. Gene-specific primers were designed using Primer v5.0 (Table S2). The normalized *eEF1Bα2* gene(BraA10g020830.3C.gene) was used as an internal control of gene expression [27]. The quality-assured RNA was used as template, with the synthesis of cDNA processed by a Takara PrimeScript RT reagent Kit (Takara, Nojihigashi, Kusatsu, Japan). The Bio-Rad CFX96 RT-PCR Detection system (Bio-Rad, Hercules, CA, USA) and SYBR Green II PCR Master mix (Takara) were used for the qRT-PCR reactions. The gene expression data were analyzed using the 2^−ΔΔCt^ method [28]. SPSS v19.0 (SPSS, Chicago, IL, USA) was used to conduct the one-way analysis of variance (ANOVA) with Duncan’s multiple range test using a significance threshold of *p* < 0.05. The results were visualized using SigmaPlot v10.0 (Systat Software Inc., San Jose, CA, USA).

## 3. Results

### 3.1. Confirmation of Investigation Periods

**Figure 1:**
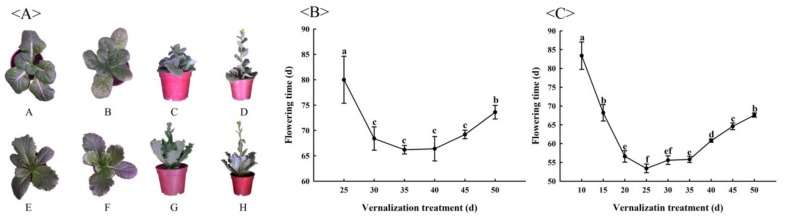
(**A**) The appearance of ‘Jin Wawa’ (JWW) (A–D) and ‘Xiao Baojian’ (XBJ) (E–H). (A), (E) Before vernalization (0 DAT); (B), (F) during vernalization (25 DAT); (C), (G) bolting; (D), (H) flowering. (**B**) The number of flowering time promoted by 4 °C vernalization treatment between ‘JWW’ and (**C**) ‘XBJ’. Values represent the means of five replicates ± standard error (SE) in the different vernalization periods. Values with the same letter are not significantly different at the *p* < 0.05 level.

From the beginning of vernalization to the end of vernalization, the number of leaves of ‘JWW’ is intuitively greater than that of ‘XBJ’, which may be related to the degree of vernalization resistance (Figure 1A). At the same time, according to its own bolting characteristics, it can be seen that at least 25 days of vernalization can promote faster flowering of ‘JWW’, whereas ‘XBJ’ only needs 10 days of vernalization to promote flowering (Figure 1B,C). For the comprehensive analysis of the differences in flowering times between the two *B. rapa* accessions, 14 different DAT [J1 (0 DAT), J2 (25 DAT), J3 (30 DAT), J4 (35 DAT), J5 (45 DAT), J6 (50 DAT), JCK (35 DAT at 25 °C), X1 (0 DAT), X2 (10 DAT), X3 (15 DAT), X4 (25 DAT), X5 (40 DAT), X6 (50 DAT), XCK (25 DAT at 25 °C), J for ‘JWW’, X for ‘XBJ’] were selected for RNA sequencing (total of 42 samples). In ‘JWW’, RNA sequencing was selected based on the following criteria: (1) underwent at least 25 DAT before flowering; (2) 35 DAT promoted ‘JWW’ flowering relatively quickly; (3) according to the difference in flowering times near 35 DAT, 30 and 45 DAT, these times were selected as representatives; (4) 50 DAT was the maximum treatment time; (5) 0 and 35 DAT at 25 °C were used as the controls. In ‘XBJ’, the criteria for RNA sequencing were as follows: (1) underwent at least 10 DAT before flowering; (2) 25 DAT promoted ‘XBJ’ flowering relatively quickly; (3) according to the difference in flowering times near 25 DAT, 15 and 40 DAT, these times were selected as representatives; (4) 50 DAT was the maximum treatment time; (5) 0 and 25 DAT at 25 °C were used as the controls.

### 3.2. DEG Analysis of ‘JWW’ and ‘XBJ’

The two *B. rapa* accessions were analyzed across other periods using 0 DAT as the control for each period. Illumina RNA-Seq analysis of the 42 samples produced 1,949,168,044 total reads and 291.28 Gb clean data. The Q30 was ≥ 93.51% and the average GC content was 47.36% (Table S3A). After processing the reads, 974,584,022 clean reads were obtained and sequence aligned with the designated *B. rapa* reference genome with an alignment efficiency ranging from 84.62% to 93.33%. The unique map ratio ranged from 77.98% to 90.88%, and the multiple map ratio ranged from 2.07% to 6.65% (Table S3B). Comparison with the *B. rapa* reference genome, 44,799 known genes and 2,280 new genes were identified.

**Figure 2:**
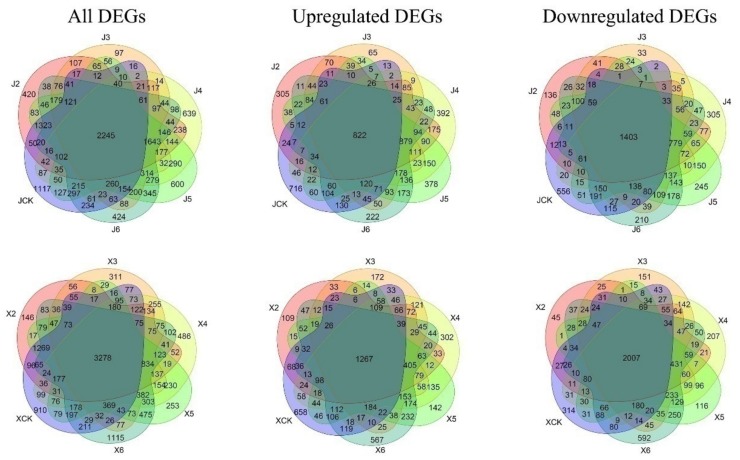
Venn diagrams of differentially expressed genes (DEGs) in the two *B. rapa* accessions during different vernalization periods. J2, J3, J4, J5, J6, and JCK represent the number of DEGs obtained by comparing ‘JinWawa’ 0 days after treatment (DAT) with 25, 30, 35, 40, and 50 DAT, and 35 DAT 25 °C, respectively. X2, X3, X4, X5, X6, and XCK represent the number of DEGs obtained by comparing ‘Xiao Baojian’ 0 DAT with 10, 15, 25, 40, and 50 DAT and 25 DAT 25 °C, respectively. False discovery rate (FDR) < 0.01 and |log2FC| > 1 were used as cut-off criteria for significance.

Multiple groups were differentially analyzed using 0 DAT (J1 and X1) as the controls to identify vernalization-related DEGs (Figure 2). In ‘JWW’, 2245 common DEGs were continuously differentially expressed (822 upregulated; 1,403 downregulated) compared to J1. Fast flowering promoted at 35 DAT in ‘JWW’ uncovered 639 DEGs (392 upregulated; 305 downregulated). In ‘XBJ’, a total of 3278 DEGs (1267 upregulated; 2007 downregulated) were continuously expressed at 10, 15, 25, 40, and 50 DAT. A total of 486 DEGs (302 upregulated; 207 downregulated) were identified at 25 DAT, which promoted fast flowering in ‘XBJ’. Clearly, ‘XBJ’ possessed more continuously expressed genes during vernalization than ‘JWW’.

**Figure 3:**
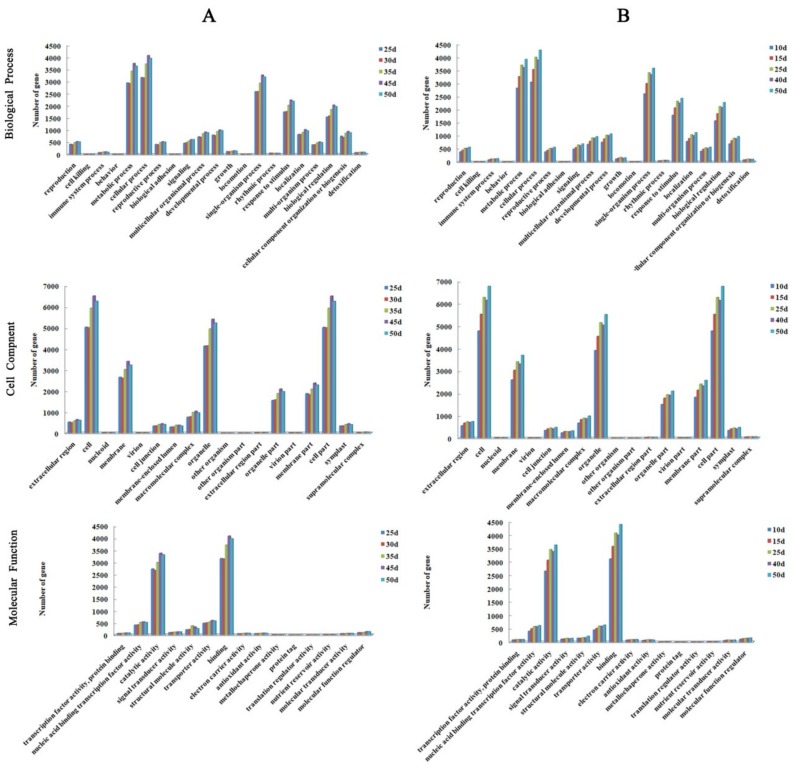
Gene ontology (GO) classification of the DEGs in ‘JWW’ (**A**) and ‘XBJ’ (**B**).

To determine the functional distribution of unigenes, GO annotation was used to classify the DEGs generated by ‘JWW’ and ‘XBJ’ during vernalization (Figure 3). The two *B. rapa* accessions exhibited a high degree of similarity in terms of unigene distribution. In the biological process category, unigenes were concentrated in the “metabolic process”, “cellular process”, and “single-organism process”. In the cell component category, most unigenes were related to “cell”, “organelle”, and “cell part”. In the molecular function category, unigenes were highly related to “catalytic activity” and “binding”. These highly enriched GO terms indicated the basic regulatory and metabolic functions of the two *B. rapa* accessions during vernalization. For the metabolic processes in the biological category, we selected the vernalization periods common to ‘JWW’ and ‘XBJ’ for comparison, which are 0 DAT (J1 and X1), 25 DAT (J2 and X4), and 50 DAT (J6 and X6). Compared to 0 DAT, we found that the change in 25 DAT and 50 DAT expression was larger in ‘XBJ’ than in ‘JWW’. Similarly, the catalytic activity-related genes in molecular functions are more pronounced in ‘XBJ’. J2 and J6 are enriched in this term by 2723 and 3303 DEGs, respectively, compared with J1.X4 and X6 are enriched in this term with 3461 and 3630 DEGs, respectively, compared with X1. Overall, in these three GO analysis categories, the expression of ‘XBJ’ in many terms has changed more than ‘JWW’.

### 3.3. Analyses of Dynamic Gene Expression Patterns during Vernalization 

**Figure 4:**
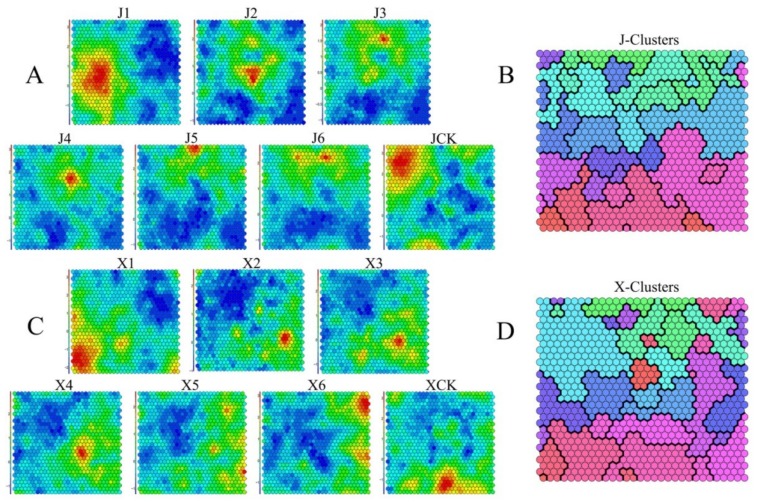
The temporal expression patterns of 21,035 DEGs in ‘JWW’ during vernalization (**A**); self-organizing feature map (SOM) clustering identified 20 clusters in ‘JWW’, in which different colors represent separate clusters (**B**); the temporal expression patterns of 21,035 DEGs in ‘XBJ’ during vernalization (**C**); SOM clustering identified 20 clusters in ‘XBJ’, in which different colors represent separate clusters (**D**). The red to blue gradient indicated upregulation and downregulation, respectively (A,C).

To determine the main transcriptional dynamics related to vernalization of the two *B. rapa* accessions, SOM was used to identify the dynamic changes of the 21,035 DEGs in different vernalization periods, as well as a cluster analysis, which was performed on multiple groups of expression patterns. The expression of normalized DEGs in each period is represented by different colors of each hexagon in the square; the red to blue gradient indicated upregulation and downregulation, respectively (Figure 4A,C). Each DEG that exhibited a similar expression level was clustered into agrid and grids with the same color from each cluster were grouped together (Figure 4B,D). The characteristic values of the gene expression levels of each grid were extracted; 20 clusters were defined according to the number of grids (Figures S1 and S2). Then, GO classification analysis was performed on each cluster to analyze the regulated DEGs of the two *B. rapa* accessions during vernalization in the three GO categories (Tables S4 and S5). Clusters that were notably regulated during vernalization were selected to analyze the differential expression patterns of the two *B. rapa* accessions.

In clusters 7, 10, 11, 13, and 15 of ‘JWW’, comparing 0 DAT and 35 DAT 25 °C, DEGs exhibited a state of continuous induction during vernalization. These genes were upregulated starting at 25 DAT and continued through 50 DAT. These continuously upregulated genes possessed biologically important functions and were used for studying the sensitivity of ‘JWW’ to vernalization. GO annotations were used for classifying the continuously increasing DEGs. Cellular components were enriched in genes related to extracellular space, lysosome, nucleolus, and endosome. In the biological processes category, DEGs were mainly enriched in regulation of gene expression and epigenetic. In the molecular functions category, these clusters had different DEGs that were enriched. Specifically, receptor activity-related genes were enriched in clusters 10, 13, and 15. Clusters 7 and 11 were enriched in genes related to enzyme regulator activity. The genes related to binding, including oxygen and lipid binding, were enriched in each of the five clusters. In clusters 3, 12, and 19 of ‘JWW’, the DEGs remained stable and exhibited low expression levels at 25 DAT. Cluster 12 reached its lowest level at 50 DAT. The GO analysis revealed that genes related to extracellular space, nucleolus, and endosome in the cellular components category were enriched in these three clusters. Cluster 3 was enriched in genes involved in the regulation of gene expression and epigenetic in the biological processes category. The molecular functions category was enriched in activity, including receptor, nuclease, and enzyme regulator activities, and binding, including oxygen and lipid binding, genes in these three clusters.

In ‘XBJ’ at 10 DAT, clusters 7 and 10 were compared between 0 DAT and 25 DAT 25 °C, revealing that the genes were continuously upregulated. The GO analysis revealed that these genes were enriched in peroxisome and nucleoplasm in the cellular components category, cellular homeostasis in the biological processes category, and lipid binding in the molecular functions category; related genes were all enriched in these two clusters. Additionally, signal transducer activity and carbohydrate binding-related genes in the molecular functions category were enriched in cluster 10 of ‘XBJ’. During the vernalization of ‘XBJ’, clusters 1, 2, and 8 exhibited stable or low gene expression and were enriched in nucleoplasm of the cellular components category. During the vernalization of ‘XBJ’, clusters 1, 2, and 8 with stable or low gene expression were enriched in nucleoplasm of the cellular components category. Meanwhile, related genes of cluster 2 were enriched in golgi apparatus, and related genes of cluster 8 were enriched in nuclear envelope. Among the biological processes category, only cluster 8, which contained low-expressing genes, was abundant in cellular homeostasis. Moreover, low-expressed genes were enriched only in cellular homeostasis. The molecular functions category was enriched in low-expressing genes related to signal transducer activity and lipid binding in clusters 1, 2, and 8.

To further understand these continuously upregulated and downregulated genes in the enrichment pathways, these clusters were analyzed by a KEGG pathway analysis (Tables S6 and S7). In ‘JWW’, cluster 7 possessed continuously upregulated genes that were notably enriched in glycolysis/gluconeogenesis, starch and sucrose metabolism, and amino sugar and nucleotide sugar metabolism. In cluster 15, the DEGs of three aspects, including proteasome, plant hormone signal transduction, and glycolysis/gluconeogenesis, were notably expressed. Notably, during vernalization, these continuously upregulated clusters were considerably enriched in the plant hormone signal transduction and starch and sucrose metabolism pathways. Moreover, the plant hormone signal transduction and starch and sucrose metabolism pathways in clusters 3, 12, and 19 were continuously considerably downregulated. Analysis of the ‘XBJ’’s gene clusters that were continuously upregulated and downregulated during vernalization revealed that the plant hormone signal transduction and starch and sucrose metabolism pathways were also considerable. Therefore, a detailed analysis was performed on the related gene expression profiles of the plant hormone signal transduction and starch and sucrose metabolism pathways. Additionally, in order to fully explain the effect of vernalization on the two *B. rapa* accessions, the photoperiod, circadian clock, and vernalization pathways were incorporated in the analysis.

### 3.4. DEGs of the Plant Hormone-Related Signal Transduction Pathway

**Figure 5:**
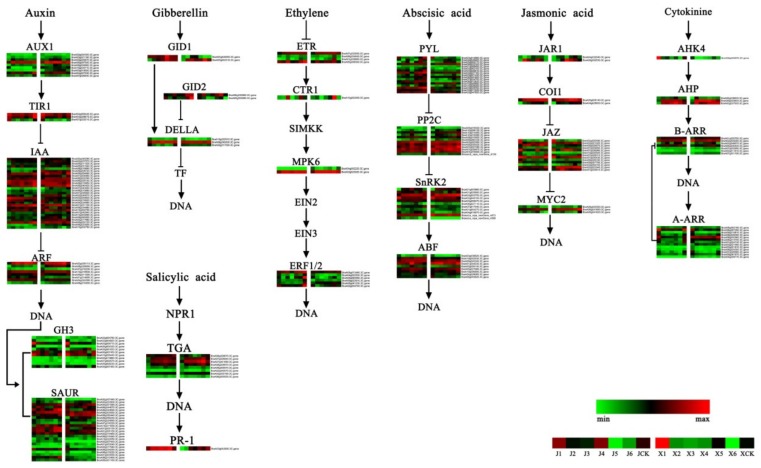
Heat map of plant hormone-related gene expression levels. Data were obtained using the log_2_fragments per kilobase of exon per million fragments mapped (FPKM) of each gene. Red and green represent up- and downregulated genes, respectively. AUX1 = auxin transporter-like protein; TIR1 = protein transport inhibitor response 1; IAA = auxin-responsive protein IAA; ARF = auxin response factor; GH3 = indole-3-acetic acid-amido synthetase GH3; SAUR = auxin-responsive protein SAUR; GID1 = gibberellin receptor GID1; GID2 = gibberellin receptor GID2; DELLA = DELLA protein RG; NPR1 = nonexpresser of PR genes 1; TGA = transcription factor TGA; PR-1 = pathogenesis-related protein 1; ETR = ethylene receptor; CTR1 = serine/threonine-protein kinase CTR1; SIMKK = SIMK kinase; MPK = mitogen-activated protein kinase; EIN2 = ethylene-insensitive protein 2; EIN3 = ethylene-insensitive protein 3; ERF = ethylene-responsive transcription factor; PYL = abscisic acid receptor PYL; PP2C = protein phosphatase 2C; SnRK2 = serine/threonine-protein kinase SRK2; ABF = abscisic acid-insensitive; JAR1 = jasmonic acid-amido synthetase JAR1; COI1 = coronatine-insensitive protein 1; JAZ = protein TIFY; MYC2 = transcription factor MYC; AHK4 = histidine kinase 4; AHP = histidine-containing phosphotransfer protein; A/B-ARR = two-component response regulator ARR.

The plant hormone signal transduction pathways exhibited different DGEs levels during the two *B. rapa* accessions vernalization. To identify the hormone similarities and differences of the two different bolting *B. rapa* accessions, the expression levels of the hormone-related DEGs were further analyzed (Figure 5).

A total of 206 DEGs related to plant hormone signal transduction were identified in the two *B. rapa* accessions (Table S8). DEGs related to the plant hormone signaling pathway of ‘JWW’ and ‘XBJ’ were enriched at various periods. During the transition from J1 (0 DAT) to J4 (35 DAT) in ‘JWW’, 44 and 40 of the 84 auxin-related DEGs were notably upregulated and downregulated, respectively. Among these 84 DEGs, 4 auxin transporter-like (*AUX1*), 9 auxin-responsive (*IAA*), 3 auxin response factor (*ARF*), 2 indole-3-acetic acid-amidosynthetase GH3 (*GH3*), and 6 auxin-responsive protein SAUR family genes (*SAURs*) were considerably upregulated (log2(J4_FPKM_/J1_FPKM_) > 1). Moreover, 2 *AUX1*, 1 protein transport inhibitor response 1 (*TIR1*) (BraA07g025310.3C), 3 *IAA*, 1 *ARF*, 3 *GH3*, and 9 *SAUR* family genes were downregulated (log2(J4_FPKM_/J1_FPKM_) < −1). Interestingly, the upregulated and downregulation of these genes had the same change in J4 versus JCK. In ‘XBJ’, 7 and 1 *AUX1*, 14 and 5 *IAA*, 4 and 1 *ARF*, 4 and 4 *CH3*, and 3 and 13 *SAUR* family genes were upregulated (log2(X4_FPKM_/X1_FPKM_) > 1) and downregulated (log2(X4_FPKM_/X1_FPKM_) < −1), respectively, in the transition from X1 (0 DAT) to X4 (25 DAT). These genes also had the same trend in X4 versus XCK. Seven DEGs were considerably expressed in the GA pathway. The two GA receptor *GID1s* were upregulated from J1 to J5 (0 to 45 DAT) in ‘JWW’, but clearly downregulated in J6 (50 DAT). In ‘XBJ’, the expression of *GID1* genes varied throughout vernalization. Expression of the 2 *GID2* genes in ‘JWW’ exhibited a fluctuating state of falling, rising, then falling and rising once more. Nevertheless, these levels rose after ‘XBJ’ showed a decline. The three DELLA protein RG (*DELLA*) genes exhibited the same trend in both accessions. Two of the four ethylene response sensor (*ETR*) genes were upregulated and two were downregulated in the transition from J1 to J4 in the ethylene (ET) pathway of ‘JWW’. In J4 versus JCK, all four *ETRs* were downregulated. In ‘XBJ’, among the four *ETRs*, X4 versus X1 and X4 versus XCK only have one *ETR* downregulated and three upregulated. Notably, a mitogen-activated protein kinase 6 (*MPK6*) gene (BraA04g002220.3C.gene) was expressed in trace amounts in ‘JWW’, or was almost absent, but decreased considerably in the vernalization of ‘XBJ’. ET-responsive transcription factor (*ERF1/2*) genes also behaved slightly differently in the *B. rapa* accessions. Compared to JCK, only one of the 17 abscisic acid (ABA) receptor *PYL* genes in the ABA pathway of ‘JWW’ was considerably upregulated in J4. In J4 versus J1, there were four *PYL* upregulated. Only one *PYL* (BraA10g000590.3C) remained the same upregulated level in both comparisons. However, compared to XCK, these *PYL* genes were upregulated and downregulated in ‘XBJ’, respectively, which also occurred in X4 versus X1. Protein phosphatase 2C (*PP2C*) genes and serine/threonine-protein kinase (*SnRK2*) genes exhibited more consistent trends during vernalization. In ‘JWW’ compared to JCK at J4, most *PP2C* genes were considerably upregulated. Compared to XCK and X4, most *PP2C* genes were considerably downregulated in ‘XBJ’. In the jasmonic acid (JA) pathway, 20 DEGs were identified. In the cytokinin (CK) pathway, 24 DEGs were identified, including 1 histidine kinase 4 X4 (*AHK4*), 3 histidine-containing phosphotransfer protein (*AHP*), and 2 two-component response regulator ARR-B family (*B-ARR*), and 13 two-component response regulator ARR-A family (*A-ARR*) genes. In the salicylic acid (SA) pathway, the expression patterns of the eight transcription factor *TGA* genes were slightly different between the two accessions, which were more conspicuous in ‘XBJ’.

### 3.5. Analysis of Starch and Sucrose Metabolism Pathways during Vernalization

**Figure 6:**
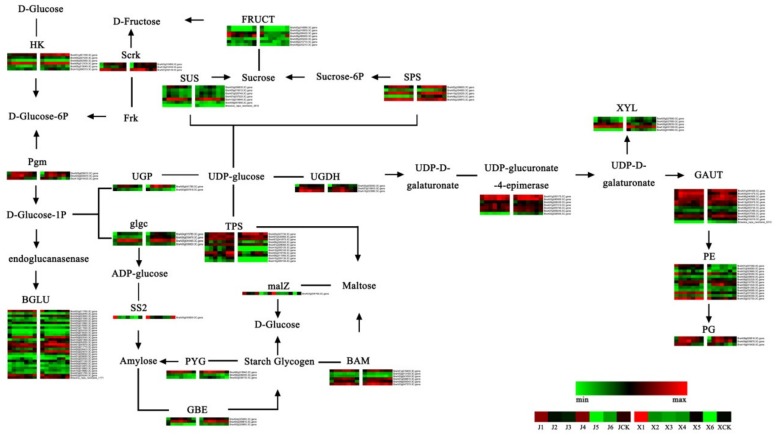
Heat map of the starch and sucrose-related gene expression levels. Data were obtained using the log_2_FPKM of each gene. Red and green represent up- and downregulated genes, respectively. HK = histidine kinase; BGLU = beta-glucosidase; pgm = phosphoglucomutase; Scrk = fructokinase; SUS = sucrose synthase; UGP = UTP-glucose-1-phosphate uridylyltransferase; UDP = uridine diphosphate–glucose; UGDH = UDP-glucose 6-dehydrogenase; malZ = alpha-glucosidase; glgc = glucose-1-phosphate adenylyltransferase large subunit; SS2 = starch synthases 2; PYG = alpha-glucan phosphorylase; GBE = glucan-branching enzyme; Frk = fyn related Src family tyrosine kinase; BAM = maltose beta-amylase; XYL = beta-D-xylosidase; GAUT = beta-D-xylosidase; PE = pectinesterase/pectinesterase inhibitor; PG = phosphoglucomutase; TPS = trehalose-phosphate synthase; FRUCT = beta-fructofuranosidase; SPS = sucrose-phosphate synthase.

The enrichment pathways investigated by the KEGG pathway analysis included the starch and sucrose metabolism pathways, which were enriched in clusters 2, 7, and 10 of ‘JWW’, and in clusters 1, 2, and 10 of ‘XBJ’. Subsequently, 134 DEGs in these pathways were further analyzed (Figure 6; Table S9). In the sucrose synthesis pathway, sucrose synthase (*SUS*) genes were notably expressed during vernalization in ‘JWW’ and ‘XBJ’, and its expression levels increased at first then decreased. However, ‘JWW’ did not express the newly discovered *SUS* gene (newGene_3912). Of the five sucrose-phosphate synthase (*SPS*) genes from J1 to J4, three were considerably upregulated (log2(J4_FPKM_/J1_FPKM_) > 1), and one were considerably downregulated (log2(J4_FPKM_/J1_FPKM_) < −1) in JCK versus J4. In ‘XBJ’ from X1 to X4, 4 were considerably upregulated (log2(X4_FPKM_/X1_FPKM_) > 1) and 1 was slight downregulated. The alpha-glucan phosphorylase (*PYG*) and 1,4-alpha-glucan-branching enzyme (*GBE*) genes in the starch synthesis pathway were notably different between the two *B. rapa* accessions. Expression of the three *PYG* and three *GBE* genes in each vernalization period of ‘XBJ’ were expressed at higher levels than ‘JWW’. Vernalization promoted the upregulation of four maltose beta-amylase (*BAM*) genes and downregulation of two genes. However, in the same period, five *BAMs* were considerably higher than JCK in ‘JWW’ at J4. In X4, three *BAM* genes were expressed at higher levels in ‘XBJ’ than XCK. Alpha,alpha-trehalose-phosphate synthase (*TPS*) genes, which play important roles in sucrose and starch synthesis, were enriched in both accessions. Four *TPSs* in ‘JWW’ were considerably upregulated (log2(J4_FPKM_/J1_FPKM_) > 1) and three were considerably downregulated (log2(J4_FPKM_/J1_FPKM_) < −1) from J1 to J4. It is worth noting that in J4 versus JCK, only two *TPSs* were considerably upregulated (log2(J4_FPKM_/J1_FPKM_) > 1) and seven were considerably downregulated (log2(J4_FPKM_/J1_FPKM_) < −1). Unlike ‘JWW’, only seven *TPSs* were expressed, three of which were considerably upregulated (log2(X4_FPKM_/X1_FPKM_) > 1) in X1 to X4 of ‘XBJ’. Only one *TPS* was considerably upregulated (log2(X4_FPKM_/X1_FPKM_) > 1) in X4 versus XCK. Two of the three phosphoglucomutase (*pgm*) genes increased and then decreased during vernalization. Two UTP--glucose-1-phosphate uridylyltransferase (*UGP*) genes, which play roles in the intermediate regulation of sucrose and starch synthesis, were expressed. Specifically, *UGP1* was continuously downregulated and *UGP2* was steadily upregulated as vernalization progressed, which was more evident in ‘XBJ’. These results indicated that the starch and sucrose metabolism pathways play important roles in regulating the responses of *B. rapa* to vernalization.

### 3.6. DEGs of the Photoperiod and Circadian Clock Pathways

**Figure 7:**
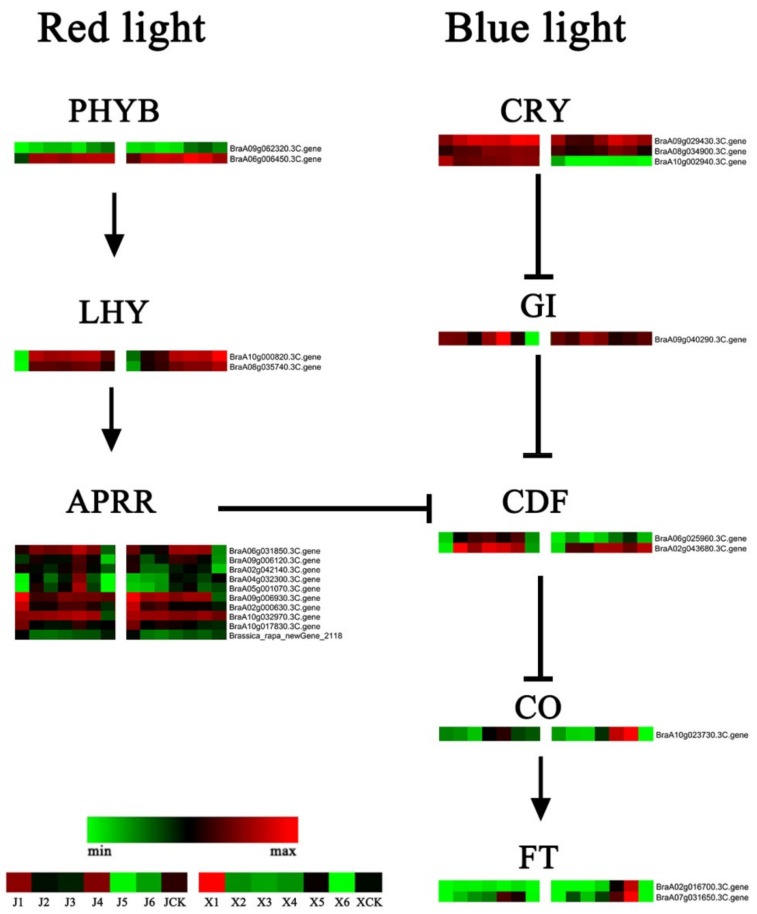
Heat map of the photoperiod and circadian clock gene expression levels. Data were obtained using the log_2_FPKM of each gene. Red and green represent up- and downregulated genes, respectively. PHYB = phytochrome B; LHY = protein LHY; APRR = two-component response regulator; CRY = cryptochrome-2; GI = gigantea; CDF = cyclic dof factor; CO = constants; FT = floweing locus T.

The photoperiod and circadian clock pathway-related genes are often referred to as flowering-related genes. The two pathways of the red and blue light absorbing plants were compared and analyzed to the 23 flowering-related genes identified in this study (Figure 7; Table S10). In ‘JWW’ from J1 to J4, 12 genes were considerably upregulated (log2(J4_FPKM_/J1_FPKM_) > 1). In ‘XBJ’ from X1 to X4, only 7genes were considerably upregulated (log2(X4_FPKM_/X1_FPKM_) > 1) and 6were considerably downregulated (log2(X4_FPKM_/X1_FPKM_) < −1). Phytochrome B(*PHYB*), two-component response regulator (*APRR*), and protein LHY (*LHY*) genes in the red-light pathway that positively regulate flowering locus T (*FT*) between the *B. rapa* accessions was slightly different during vernalization. It was worth noting that the two phytochrome B (*PHYB*) genes upregulated in J4 versus J1 were downregulated in J4 versus JCK. Ten *APRRs* were up- and downregulated during vernalization; the expression levels of *APRRs* in ‘JWW’ were generally higher than ‘XBJ’. The expression of *LHYs*, which play intermediate roles in the red-light pathway, were not notably different between the two accessions. The cryptochrome-2 (*CRY*), gigantea (*GI*), and cyclic dof factor (*CDF*) genes in the blue light pathway were negatively regulated. *CDFs* were also regulated by red light-related genes. In the comparison between J4 and JCK, the *GI* and *CDF* genes in ‘JWW’ were upregulated during vernalization. Comparing X4 to XCK, vernalization slightly upregulated the *GIs* of ‘XBJ’, but downregulated *CDFs*. ‘XBJ’ also upregulated flowering genes, including zinc finger protein constants (*CO*) and *FTs*, during vernalization at higher levels than ‘JWW’. These results indicated that the photoperiod and circadian clock pathways may play important roles in regulating *B. rapa* flowering during vernalization.

### 3.7. Identification of VernalizationPathway DEGs

**Figure 8:**
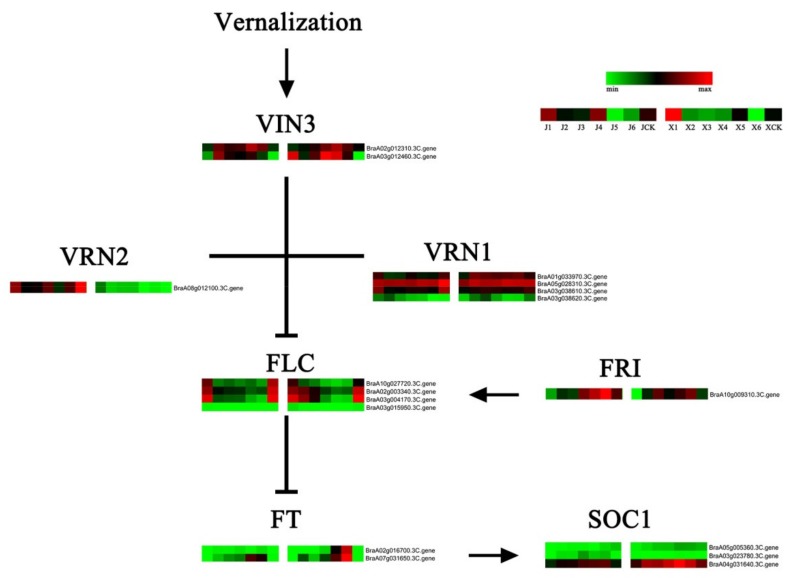
Map of vernalization-related gene expression levels. Data were obtained using the log_2_FPKM of each gene. Red and green represent up- and downregulated genes, respectively. VIN3 = vernalization insensitive 3; VRN1 = B3 domain-containing transcription factor VRN1; VRN2 = B3 domain-containing transcription factor VRN2; FLC = flower locus C; FRI = frigida; SOC1 = suppressor of constans overexpression 1.

Studies on the molecular mechanism of vernalization in dicotyledons have focused on the expression changes of *FLC* and its related regulatory genes. In this study, *FLCs* and their related vernalization regulating genes were identified (Figure 8; Table S11). *VIN3*, *VRN2*, and *VRN1* genes were found to be negative regulators of *FLC*. Moreover, vernalization promoted the upregulation of *VIN3*s. The expression patterns of *VRN1* between the two *B. rapa* accessions exhibited contrasting trends. Moreover, *VRN2* did not change much during vernalization. Vernalization reduced all four *FLCs* and the reductions in ‘XBJ’ were slightly greater than ‘JWW’. *FRI* positively regulates *FLC* [10], but was not affected by vernalization. It can be observed that *FRI* was not very different between X4 and XCK, and there was not much difference in *FRI* expression between J4 and JCK. Although *FRI* expression in both accessions continuously increased as vernalization progressed, it could not be explained by the effects of vernalization. Downregulated *FLCs* negatively affected the upregulation of *FTs*, which promoted the upregulation of the flowering factor, suppressor of constans overexpression 1 (*SOC1*). *SOC1* in ‘XBJ’ was upregulated more than ‘JWW’.

### 3.8. qRT-PCRValidation of DEGs

**Figure 9:**
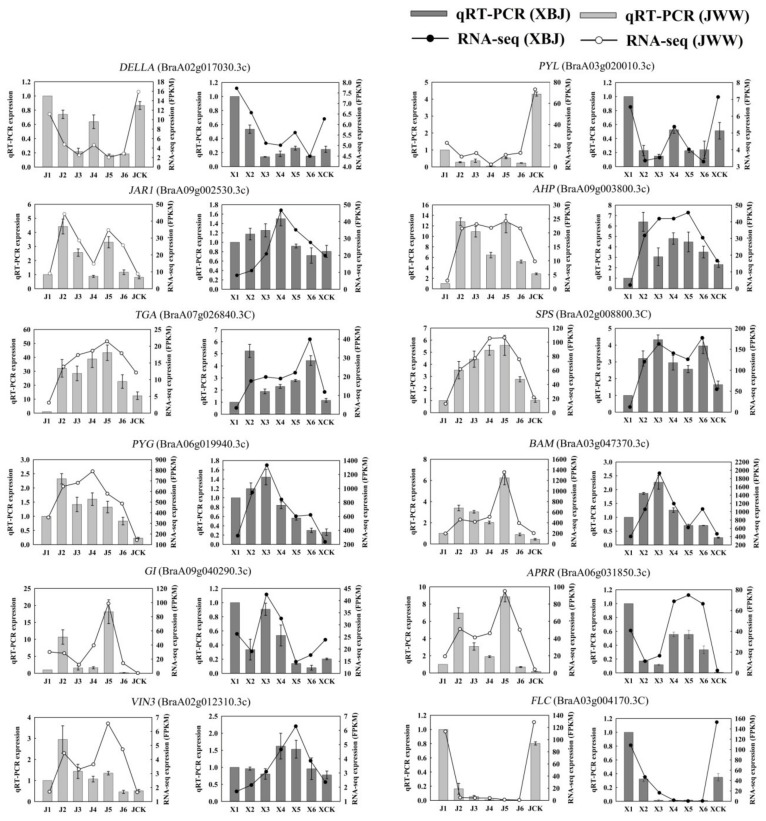
qRT-PCR was performed using 12 randomly selected DEGs. Bar and line graphs represent the qRT-PCR and RNA-Seq data, respectively. Data are presented as the mean ± standard error (SE).

To verify the results of the RNA-Seq analysis, 12 DEGs were verified using qRT-PCR, including five plant hormone signal transduction (i.e., *DELLA*, *PYL*, *JAR1*, *AHP*, and *TGA*), three starch and sucrose metabolism (i.e., *SPS*, *PYG*, and *BAM*), two photoperiod and circadian clock (i.e., *G*I and *APRR*), and two vernalization (i.e., *VIN3* and *FLC*) genes (Figure 9). The 12 DEGs exhibited the same trends between the RNA-Seq and qRT-PCR results, indicating that the transcriptome analysis was accurate (R^2^ = 0.764; Figure S3).

## 4. Discussion

Studies on transcriptome regulation based on phenotypes is a powerful research method [29,30]. Using this analytical method, DEGs and related regulatory pathways produced by Asiatic lily and tropical lotus during vernalization have been investigated [31,32]. With regard to *B. rapa*, the reference genome has been updated for several generations, and various regulatory pathways have been continuously studied [33,34]. However, only a few studies have been conducted on the vernalization control networks in *B. rapa* at different periods. Thus, in order to clarify the associated regulatory mechanisms, two bolting accessions were selected for transcriptome analysis in this study. A total of 974,584,022 clean reads and 291.28 Gb clean data were obtained. After comparison with the *B. rapa* reference genome, 44,799 known genes and 2280 new genes were identified. A SOM analysis was performed and screened 21,036 DEGs in ‘JWW’ and ‘XBJ’ (Figure 4). GO and KEGG analyses were also performed on clusters that were continuously up- and downregulated during vernalization. DEGs that were enriched in the hormone signal transduction and sucrose metabolism pathways of these continuously up- and downregulated clusters were identified. Changes in the DEGs of these pathways during vernalization in the two *B. rapa* accessions were the primary focus of this study. Moreover, DEGs in the photoperiod and circadian clock, and vernalization pathways were analyzed.

### 4.1. Changes in the Plant Hormone Signal Transduction Pathway during Vernalization

The transition from vegetative to reproductive growth is a necessary process in plant flowering and reproduction. The shoot tip meristem (SAM) is an important part of the plant that helps complete the transition from vegetative to reproductive growth. During the vegetative growth stage, leaves formed around the SAM will generate lateral meristems. Then, a series of environmental influences and induced changes in endogenous signals will change the state of the SAM and induce flowering [35]. Plant hormones play a role in the flowering time syndrome of *Arabidopsis* [36]. However, in *B. rapa*, the regulation of hormones during vernalization remains unclear. Here, a layered heat map of the 7 plant hormone pathways of the two *B. rapa* accessions during vernalization was constructed to analyze this process in detail (Figure 5, Table S8).

Auxin functions in the initiation of floral primordia and flower development [37]. *Aux/IAAs* are primarily responsive to auxin genes. Aux/IAA stability and activity are regulated by auxin [38]. The interaction between Aux/IAA and TIR1 plays a role in regulating auxin signal transduction [39]. Mutations that occur in Aux/IAA and TIR1affect the interaction, resulting in low-auxin responses [40]. Similar to carbohydrates, auxin concentrations affect flower induction in different ways. Specifically, low concentrations promote flowering, while high concentrations delay flowering [36]. In *Arabidopsis*, the local accumulation of auxin releases *ARF5* due to *Aux*/*IAA* inhibition, which subsequently promotes a rise in *CH3* levels, as well as high expression levels of *SAUR* family genes, and is the leading cause of flowering on the lateral side of the SAM [41]. In this study, ‘JWW’ and ‘XBJ’ upregulated *ARFs*, *CH3s*, and *SAUR* family genes with vernalization. These genes may be upregulated in the same way that auxin promotes flowering in Arabidopsis. This result indicates that, under the influence of vernalization, the auxin genes of the two *B. rapa* accessions may affect flower meristems, as well as their bolting and flowering abilities at different times.

GAs are a plant hormone involved in the floral transformation process of *Arabidopsis* and play a key role in controlling flowering time [2]. In *Arabidopsis*, *GID1* is a positive regulator of GA signaling. The GID1−GA complex accelerates the degradation of the *DELLA* protein [42]. In this study, two *GID1s* were upregulated in the first four vernalization periods, but notably declined in the fifth period of ‘JWW’. However, in ‘XBJ’, *GID1* levels varied depending on the vernalization stage. The three *DELLAs* exhibited the same trend in both accessions. As vernalization continued, two *DELLAs* were downregulated and one was upregulated. These results indicated that the GA signaling pathway may play a role in the transition from the growth period during vernalization of these two accessions.

ET is a gas phytohormone that participates in plant stress responses and regulates a variety of developmental processes [43]. In *Arabidopsis*, the ET receptor possesses fivemembers and has been classified based on certain characteristics into ET receptor 1 (*ETR1*) and ET receptor 2 (*ETR2*) [44]. The serine/threonine-protein kinase *CTR1* gene plays an important role in ET signal transduction [45]. There is a *MPK* module located between *CTR1* and ethylene-insensitive protein 2 (*EIN2*) [46]. A previous study demonstrated that once *MPK6* loses its function, the ET signaling pathway will return to normal, indicating that *MPK6* may not participate in this pathway [47]. However, in the two *B. rapa* accessions of this study, ‘XBJ’ exhibited a large change in two *MPK6s* during vernalization compared to ‘JWW’. Moreover, *EIN2* is the positive regulator of the ET signaling pathway [48]. *EIN3* is located downstream of *EIN2*. *EIN2* and *EIN3* work together and act on the ET-responsive transcription factor (*ERF1/2*) [49]. During vernalization, *ERF1/2* genes were differentially expressed in the two *B. rapa* accessions of this study, which may explain the different conversion flowering periods.

ABA is a plant hormone that regulates plant development [50]. PYL family proteins are receptors of the ABA signaling pathway and inhibit *PP2C* in an ABA-dependent manner [51,52]. *PP2Cs* interact with *SnRKs* and inactivate them due to phosphorylation [53]. A previous study demonstrated that ABA played an inhibitory role in flowering time by modulating *DELLA* activities in the GA pathway [54]. A separate study found that ABA may play a role in hindering flowering and affect flowering by altering *FLCs* [55]. In this study, *PYL* genes were differentially expressed in both accessions and exhibited different trends. In the fourth period of vernalization, *PYLs* in ‘JWW’ were considerably downregulated compared to the control, while the *PYLs* in ‘XBJ’ were upregulated and downregulated compared to the control. Moreover, vernalization continuously upregulated *PP2C* genes, thereby blocking the generation of some *SnRK2* genes; the difference was more noticeable in ‘XBJ’. Therefore, the ABA signaling pathway may participate in regulating different growth period transformations in these two *B. rapa* accessions.

JA is a lipid plant hormone that plays multiple roles in plant growth and development [56]. CKs are a class of classical hormones that regulate both the division cycle and meristem homeostasis [57]. SAs are involved in plant defense functions and regulate leaf senescence [58]. In this study, the genes involved with these three plant hormones changed during vernalization. jasmonic acid-amido synthetase JAR1 (*JAR1*) is one of 19 closely related *Arabidopsis* genes that issimilar to the auxin-induced soybean *GH3* gene [59]. Methyl JA (MeJA)-treated vernalization in insensitive spring wheat exhibited flowering and considerably downregulated *TaVRN1* and *TaFT1* genes, suggesting that MeJA may modulate vernalization and flowering time in wheat [60]. Previous studies have reported that CK promotes the formation of floral meristems and exogenously applied CK promotes *Arabidopsis* flowering [61,62]. The phytochrome signaling intersects with SA signaling [63], which correlates with flowering [64].

To summarize, different hormone biosynthesis and signaling pathways were differentially expressed during vernalization in the *B. rapa* accessions of this study. Clearly, this complex hormone signaling network accelerates the transition from vegetative to reproductive growth during vernalization in *B. rapa*.

### 4.2. Gene Expression Changes in the Starch and Sucrose Metabolism Pathways

The rapid propagation of plants is closely related to flowering time; carbohydrates are thought to play a crucial role in this process [65]. Sucrose and starch are the primary products of photosynthetic carbon assimilation. In lilies, sucrose treatment accelerates flowering [66]. Moreover, *SUS* is a key enzyme involved in the sucrose metabolism of plants [67], while *SPS* is a key enzyme that catalyzes the first step of the sucrose synthesis pathway [68]. ‘XBJ’ possessed a new *SUS* gene (newGene_3912), and the expression of *SPSs* in this accession were slightly higher than ‘JWW’ (Figure 6, Table S9).

Starch is the main carbohydrate stored, which allows them to meet their long-term carbon needs [69]. The main component of starch is synthesized by starch synthases (*SS2*), *GBEs*, and *PYGs* [70,71]. ‘XBJ’ highly expressed three *PYGs* and *GBEs* in each vernalization period. These differences indicated that the starch and sucrose metabolism pathways may play important roles in regulating *B. rapa* with different bolting times, but different roles in activating the SAM. Maltose is produced during the conversion of starch to sucrose in leaves under dark conditions [72]. *BAM* could potentially produce maltose as a key metabolite and osmolyte via starch hydrolysis [73]. Maltose is the primary source of carbon during cellular metabolism and growth at night [74]. Four *BAMs* were upregulated during vernalization in the two *B. rapa* accessions. However, when comparing the same vernalization period to the control, five *BAMs* were upregulated in ‘JWW’ and three in ‘XBJ’. These results indicated that maltose may provide the two *B. rapa* accession with sufficient energy required for normal growth during vernalization at night.

In *Arabidopsis*, *TPS* plays a critical role in controlling the transition from vegetative to reproductive growth [75,76]. *TPS* catalyzes the formation of trehalose-6-phosphate (*T6P*) from glucose-6-phosphate and uridine diphosphate–glucose (UDP-glu) and is a signaling molecule that relays information regarding carbohydrate availability to other signaling pathways [77,78]. The loss of *TPS* resulted in extremely delayed flowering in *Arabidopsis*. The *TPS* pathway also acts as a signal that coordinates the induction of flowering by regulating the expression of key floral integrators in the leaves and SAM [79]. In this study, the *TPSs* of ‘XBJ’ expressed during vernalization were less than ‘JWW’. It may be that the late-bolting ‘JWW’ accession requires higher *TPS* expression in order to promote faster flowering. The upregulation of *pgms* promotes the production of polysaccharides, which has been demonstrated in mushrooms [80]. In this study, *pgms* increased at first and then decreased during vernalization. Therefore, it is speculated that *pgms* rose rapidly at the beginning of vernalization in order to provide sufficient energy for normal growth, and then it gradually decreased after the vernalization transformation period. In young and mature leaves, *UGP* is primarily involved in the sucrose biosynthesis pathway, the major form of transport of carbon in plants, which provides energy to plants [81]. In this study, two *UGP* genes exhibited opposing states during vernalization, in which, one was upregulated and the other was downregulated. The underlying mechanisms for these contrasting trends, however, require further investigation.

### 4.3. Photoperiod and Circadian Clock Pathways

The photoperiod and circadian clock pathways function as a whole, as plants coordinate their metabolism according to changes in photoperiods, which thereby induces flowering. Expression changes in the *CO*/*FT* mode is the core link between the photoperiod induction pathway [3]. The purpose of this study was to analyze the influence of vernalization on the related genes in this pathway under certain photoperiods (Figure 7, Table S10). *CO* is a typical circadian clock control gene that acts on the *FT* promoter region and is the main positive regulator of *FT* [82]. The red-light pathway positively regulates *CO* and *FT*, which were continuously regulated by *CDFs* through *PHYBs*, *LHYs*, and *APRRs*. Among them, *APRRs* were considerably different between the two *B. rapa* accessions and may affect bolting. The two *PHYB*s upregulated in J4 versus J1 were downregulated in J4 versus JCK, suggesting that *PHYB*s may not be affected by vernalization in ‘JWW’. *CDFs* are intermediate regulatory genes in this pathway that are regulated by both red and blue light. Specifically, vernalization downregulated the *CDFs* of ‘XBJ’, but upregulated the *CDFs* of ‘JWW’. Thus, *CDF* may be a gene that does not affect late-bolting *B. rapa* during vernalization.

### 4.4. DEGs of the Vernalization Pathway

Land plant flower development undergoes floral induction, floral primordia, and floral organ development phases. In the flowering induction stage, although there are no morphological changes in the SAM, a series of related genes centered around *FT* undergo various changes, forming a complex regulatory network to achieve the transition from vegetative to reproductive growth [83]. In *B. rapa*, vernalization is the most important process that induces flowering. Currently, studies on the underlying molecular mechanism of the vernalization pathway in *A. thaliana* mainly focus on changes in the expression levels of *FLCs*. Among them, *BrFLC1* and *BrFLC2* of *B. rapa* respond to the regulation of vernalization and affect flowering time [84]. In this study, three of the four FLCs decreased considerably during vernalization. The decrease in ‘XBJ’ was larger than that in ‘JWW’, and it continually decreased in the first five vernalization periods (Figure 8, Table S11). *VRN1*, *VRN2*, and *VIN3* are negative regulators of *FLC,* and previous studies demonstrated that *VRN1* and *VRN2* in *Arabidopsis* were not induced by vernalization and only play a role in the persistently low expression of *FLC* at the end of vernalization [12,13,14]; thus, the results of this study were inconsistent, as two *B. rapa VRN1s* exhibited opposing modes during vernalization. However, these differences require further investigation. Changes in *VRN2* were consistent with previous results [13]. *VIN3s* were upregulated in the first five vernalization periods and downregulated in the last vernalization period. Therefore, it is speculated that *B. rapa* transitioned to reproductive growth just before the last vernalization period and, hence, the downregulation of *VIN3s*.

## 5. Conclusions

**Figure 10:**
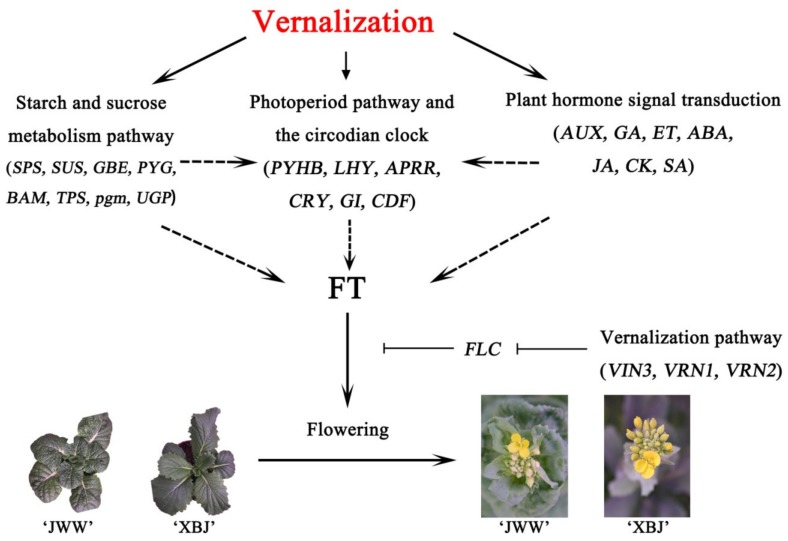
Summary of vernalization transcript regulation in *B. rapa*. Black arrows indicate promotion, and inverted T bars indicate repression. Dashed lines indicate that further research is needed.

To date, studies on the mechanisms related to vernalization of *B. rapa* have focused only on the comparison between the vernalized and unvernalized, and related research approaches have been relatively narrow. Vernalization promotes plant bolting and flowering as an important trait in productivity and reproduction; thus, it is necessary to clarify its effects on bolting and flowering in different vernalization periods. In this study, two *B. rapa* accessions with different bolting times were investigated over a series of vernalization periods. For the related flowering traits, the vernalization periods were selected for comparative transcriptome analysis to describe the effects of different vernalization periods on *B. rapa*. According to the results, the *B. rapa* accessions exhibited specific differences at each vernalization period, indicating that research on vernalization should be a process of vernalization, not a comparison between vernalized and unvernalized. In comparing the two accessions, key genes of seven hormone pathways of the plant hormone signal transduction pathway during vernalization were notably different, and the difference between late-bolting ‘XBJ’ was greater than early-bolting ‘JWW’. This difference was also confirmed in the vernalization pathway. In the starch and sucrose metabolism and photoperiod and circadian clock pathways, *TPS*, *UGP*, and *CDF* exhibited different expression patterns, but these differences require further analysis. Notably, *VIN1*, which does not play a role during vernalization in other dicotyledons, exhibited opposing expression patterns in this study, which may be a new avenue for research on vernalization in *B. rapa* in the future. The transcriptional regulation of *B. rapa* vernalization was summarized in this study (Figure 10), which provides a foundation for future investigations on the molecular mechanisms of *B. rapa* with different abstractions during vernalization.

## Supplementary Materials

The following are available online at https://www.mdpi.com/2073-4425/11/4/392/s1, Figure S1. Clusters expressions associated with ‘JWW’ vernalization periods. The x-axis represents vernalization periods, and the y-axis represents expression. Figure S2. Clusters expressions associated with ‘XBJ’ vernalization periods. The x-axis represents vernalization periods, and the y-axis represents expression. Figure S3. Linear relationships between qRT-PCR data and RNA-Seq data of related genes. Table S1. The 21035DEGs in both *B. rapa* accessions at different periods (J for ‘JWW’; X for ‘XBJ’). Table S2. Specific primers for differential gene sequences for qRT-PCR. Table S3. (A) Statistics of Illumina sequencing data (J for ‘JWW’; X for ‘XBJ’); (B) The sequencing and mapped results obtained from each sample (J for ‘JWW’; X for ‘XBJ’).Table S4. GO classification of DEGs in each clusters for the ‘JWW’. Red indicates the number of terms genes that continuously increase; blue indicates the number of terms genes that continuously decrease. Table S5. GO classification of DEGs in each clusters for the ‘XBJ’. Red indicates the number of terms genes that continuously increase; blue indicates the number of terms genes that continuously decrease. Table S6. Analysis of pathways involving up- and downregulated genes for clusters in ‘JWW’. Table S7. Anlysis of pathways involving up- and downregulated genes for clusters in ‘XBJ’.Table S8. Plant hormone related DEGs in two *B. rapa* accessions during the vernalization process. Table S9. Starch and sucrose related DEGs in two *B. rapa* accessions during the vernalization process. Table S10. Photoperiod and circadian clock related DEGs in two *B. rapa* accessions during the vernalization process. Table S11. Vernalization related DEGs in two *B. rapa* accessions during the vernalization process.
