# Supplementary figures and images for: Comparative Transcriptome Analysis of Gene Expression and Regulatory Characteristics Associated with Different Vernalization Periods in Brassica rapa
Source: Genes (Basel). 2020 Apr 5;11(4):392. doi: 10.3390/genes11040392 (PMC7231026; doi:10.3390/genes11040392)

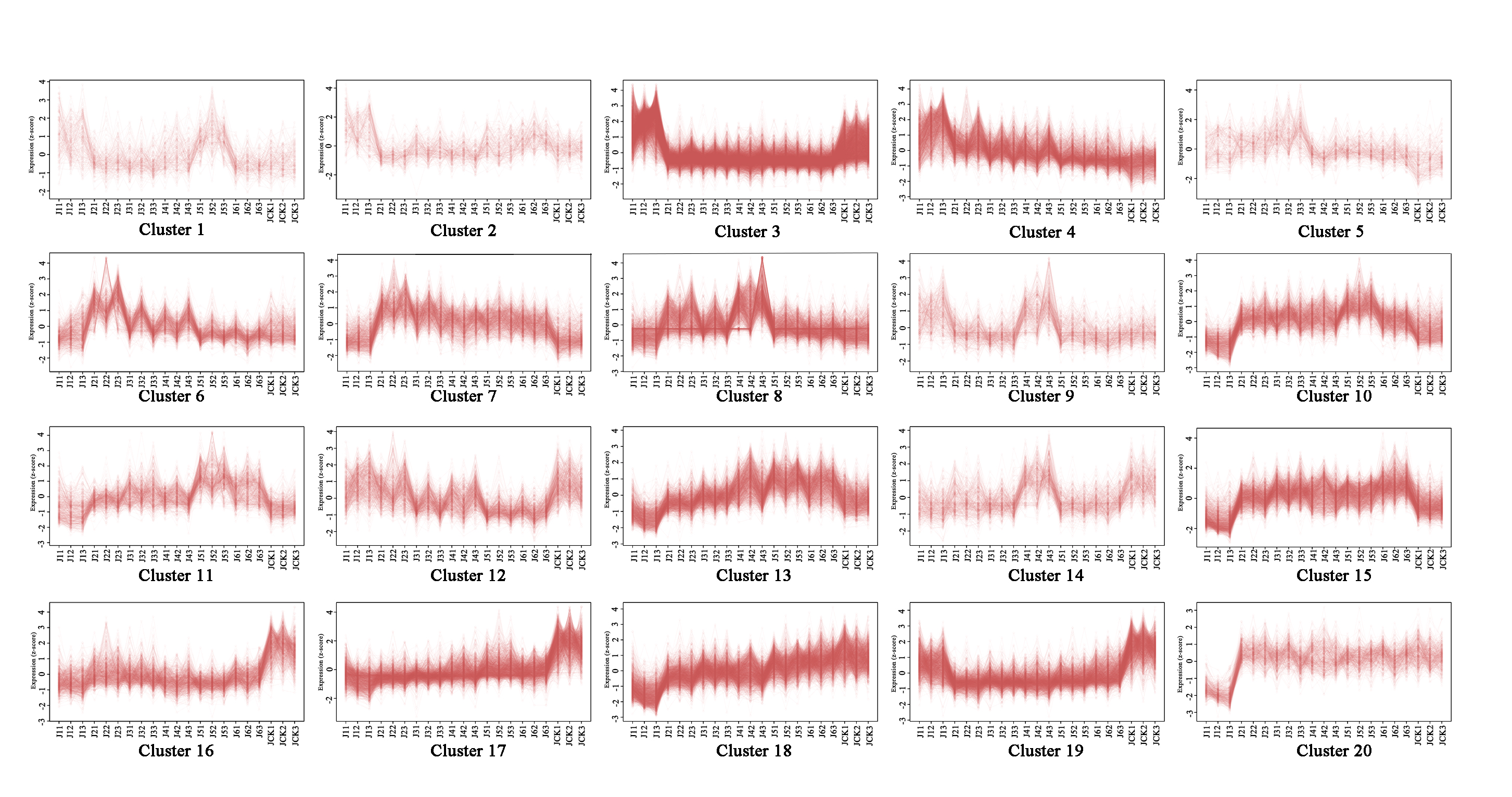

Supplement: Supplementary file 1 [file genes-11-00392-s001.zip › genes-740327-supplementary/Supplementary Materials/Figure S1.jpg]

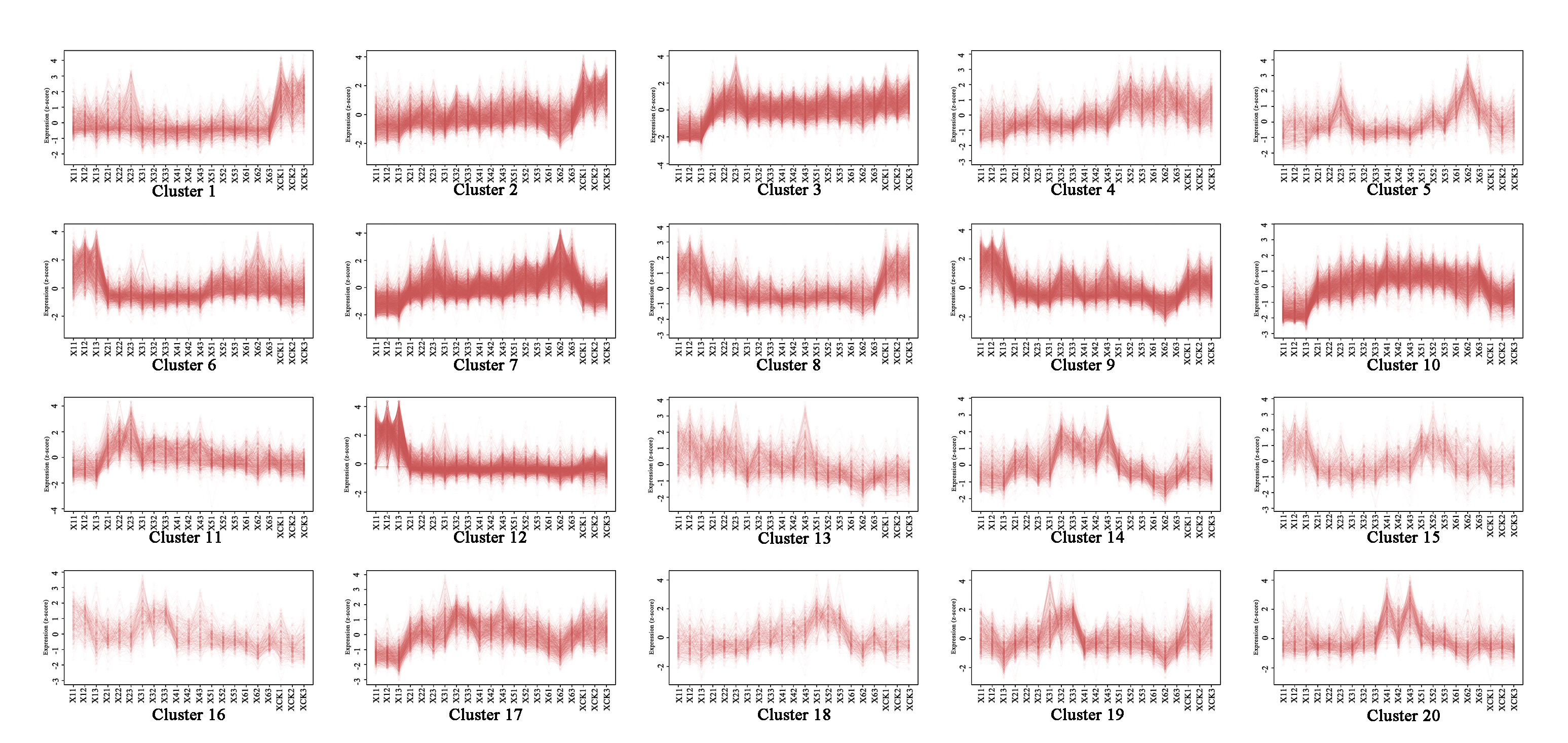

Supplement: Supplementary file 1 [file genes-11-00392-s001.zip › genes-740327-supplementary/Supplementary Materials/Figure S2.jpg]

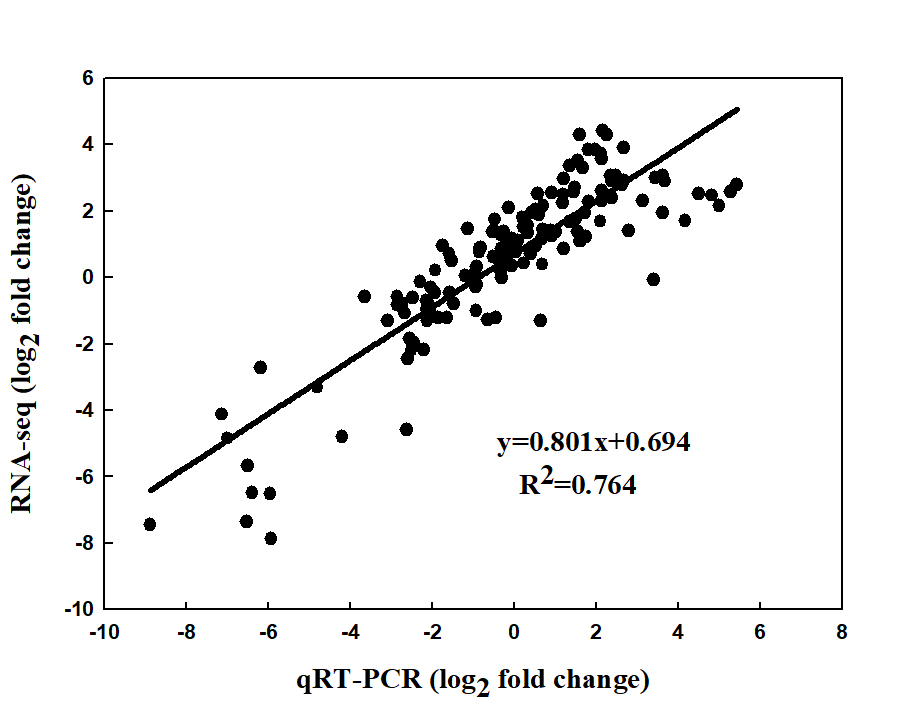

Supplement: Supplementary file 1 [file genes-11-00392-s001.zip › genes-740327-supplementary/Supplementary Materials/Figure S3.JPG]
